# Using the Multi-Theory Model (MTM) of Health Behavior Change to Explain Intentional Outdoor Nature Contact Behavior among College Students

**DOI:** 10.3390/ijerph17176104

**Published:** 2020-08-21

**Authors:** Manoj Sharma, Erin Largo-Wight, Amar Kanekar, Hana Kusumoto, Stephanie Hooper, Vinayak K. Nahar

**Affiliations:** 1 Department of Environmental & Occupational Health, School of Public Health, University of Nevada, Las Vegas, NV 89119, USA; manoj.sharma@unlv.edu; 2 Department of Public Health, University of North Florida, Jacksonville, FL 32224, USA; largo.wight@unf.edu (E.L.-W.); hana.kusumoto@gmail.com (H.K.); stephanielynnhooper@gmail.com (S.H.); 3 College of Business, Health and Human Services, University of Arkansas at Little Rock, Little Rock, AR 72204, USA; axkanekar@ualr.edu; 4 Department of Dermatology, School of Medicine, University of Mississippi Medical Center, Jackson, MS 39216, USA; 5 Department of Preventive Medicine, School of Medicine, University of Mississippi Medical Center, Jackson, MS 39216, USA

**Keywords:** perceived stress, nature contact, distress, health behavior, college students

## Abstract

Nature contact is an emerging health behavior and is defined as the interaction between human beings and animals, plants, natural scenic views, or outdoor activities. Studies have shown that exposure to the outdoors (as a means of contact with nature) reduces perceived stress and promotes health and wellbeing among varying populations in many settings. To date, however, there are few studies exploring the impact of nature contact among college students, especially in the United States. In addition, the determinants of nature contact behavior have not adequately been explored using behavioral theories. The purpose of this study was to use the multi-theory model (MTM) of health behavior change, a contemporary fourth-generation behavioral theory in explaining intentional outdoor nature contact behavior among college students. Using a cross-sectional design, 401 students completed the validated survey based on MTM. Of these, 281 met the inclusion criteria. The mean score for perceived stress based on the Perceived Stress Scale (PSS-10) in the sample was 21.60 (7.08) units, with a possible minimum and maximum scores ranging from 0 to 40 units. Constructs of behavioral confidence (standardized coefficient = 0.591, *p* < 0.001) and changes in the physical environment (standardized coefficient = 0.271, *p* < 0.001) from MTM accounted for 57.5% of the variance in the initiation for intentional outdoor nature contact behavior. All the three constructs of MTM—namely, emotional transformation (standardized coefficient = 0.173, *p* = 0.021), practice for change (standardized coefficient = 0.317, *p* < 0.001), and changes in the social environment (standardized coefficient = 0.204, *p* = 0.002)—were statistically significant and contributed substantively toward the variance (31.0%) in sustenance. MTM provides a useful and pragmatic framework for designing interventions to promote intentional nature contact behavior among college students.

## 1. Introduction

Exposure to nature has been shown to improve the quality of life for all people [1]. Contact with nature has been found to positively influence outcomes in several diseases and conditions, including depression, anxiety, attention deficit hyperactivity disorder (ADHD), cardiovascular disease, Type 2 diabetes mellitus, cancer, obesity, and mobility impairment; boost immunity in some infectious diseases; aid in recovery from surgery; improve birth outcomes; and provide relief from musculoskeletal symptoms, relief from migraines, and improvement in respiratory diseases [2,3,4,5]. Such contact with nature is especially beneficial for mental health and wellbeing [6].

Studies have shown that contact with nature impacts mental health through better stress appraisal, relaxation, and stress recovery [7,8], and improves overall health through boosted immune functioning as well as through other mechanisms, including environmental factors and altered behaviors [2]. Nature contact behavior is a significant predictor for nature connectedness [9]. A meta-analysis exploring the linkages of nature connectedness with happiness found that it had a statistically significant effect size (*r* = 0.19) [10]. Specifically, the relationship of nature connectedness was found between vitality (*r* = 0.24), positive affect (*r* = 0.22), and satisfaction with life (*r* = 0.17). Contact with nature also fosters pro-environmental attitudes and behaviors [9,11]. It is clear that contact with nature has myriad valuable effects on health and wellbeing, particularly mental health and wellbeing.

However, contact with nature has been decreasing as more people live in urban areas and spend the majority of their time indoors [12]. The problem is further compounded by excessive time spent in front of a screen, a behavior that is increasing, especially among young adults [13]. Furthermore, young adults, particularly college students, experience a plethora of stressors that cause distress in their lives. Among the common stressors are those related to academic performance, including test anxiety, the pressure to succeed, relationships, and worry about plans after graduation [14]. Stress has been strongly correlated with the occurrence of mental health disorders and suicide in college students [15]. Mental health-related illnesses generally have their onset before the age of 25 years [16]. According to World Health Organization’s survey from 21 countries, 20.3% of college students had mental disorders in the past 12 months, of which anxiety disorders were most common [17]. 

In recent years, various interventions to reduce stress have been designed and evaluated in college students. Some of these have used web-based interventions [18,19], mobile-app based mindfulness meditation [20,21], biofeedback [22], canine play [23], deep breathing [24], vestibular stimulation using swings [25], and others. These interventions have yielded mixed results in terms of efficacy and have used very small sample sizes. Very few findings used behavioral theories and, if used, there were limitations on how constructs were operationalized. Furthermore, it is notable that few of these interventions have utilized the intentional promotion of nature contact behavior as a modality for stress management, especially in the United States. A recent scoping review found 13 studies with college students, of which 10 were from Japan, apparently by the same group of researchers. The sample sizes of these studies were very small, and the explicit use of behavioral theories in promoting exposure to nature contact behavior has not been observed [26]. The determinants of nature contact behavior and its putative role in stress management among college students need to be studied. Therefore, the purpose of this study was to utilize a contemporary fourth-generation behavioral theory in explaining intentional outdoor nature contact behavior among college students. Secondarily, the study also explores the prevalence of intentional outdoor nature contact behavior among college students at a select southeastern university and examined its linkage with perceived stress. For this study, intentional outdoor nature contact behavior was defined as an interaction between human beings and animals, plants, natural scenic views, or outdoor activities.

### Theoretical Framework

The theory used in this study was the multi-theory model (MTM) of health behavior change [27,28]. According to this theory, behavior change is bisected into (1) initiation and (2) sustenance or maintenance. For initiation, the individual needs to be convinced that the advantages are more than the disadvantages (participatory dialogue), must have behavioral confidence, and must have support from the physical environment. For the maintenance of behavior change, the person must be able to transform his or her emotions into goals, continually strive for change (practice for change), and must have support from their social environment. The theory has been used in cross-sectional designs with a variety of health behaviors [29,30,31,32,33,34,35,36,37] and in experimental designs with physical activity [38], fruit and vegetable consumption [39], and water pipe cessation [40], with encouraging results. This was the first study using MTM with intentional outdoor nature contact behavior. 

## 2. Methods

### 2.1. Study Design

A cross-sectional study design was used for this study. The independent variables in this design were the constructs of the multi-theory model (MTM) of health behavior change for each of the two models: initiation and sustenance. For the initiation model, the dependent variable was the intent for practicing intentional outdoor nature contact for 20 min daily in the upcoming week. For the sustenance model, the dependent variable was the intent for practicing intentional outdoor nature contact for 20 min daily for the next five years. The rationale for choosing the dose of 20 min for this study seems justified based on the findings of a scoping review [26].

### 2.2. Population and Sample

The target population for this study was undergraduate students. The study sample was recruited at a southeastern university in the United States. The participants from the target population for this study (3494 undergraduate college students) were identified and recruited via the University Institutional Research Office. The number of participants to be recruited for this study was based on a power analysis using G*Power [41]. In calculating the sample size, the alpha was set as 0.05 and the power at 0.80, the number of predictors in each model was three, and the effect size was estimated to be 0.05 (small). These assumptions yielded a quota sample size of 222, which was considered sufficient for this study. An online survey was sent via an online survey tool along with two reminder survey invitations to the student pool. The quota sample size was met at the end of the second reminder. Respondents that met the following eligibility criteria were included: (1) aged 18 years or older; (2) during the past 24 h, they did not engage in intentional outdoor nature contact for stress reduction; and (3) over the past 7 days, they practiced less than 20 min of intentional outdoor nature contact for stress reduction on average each day.

### 2.3. Instrumentation 

For this study, nature contact was defined as the interaction between human beings and animals, plants, natural scenic views, and outdoor activities. Specifically, intentional outdoor nature contact was reified and was defined as spending time outdoors to reduce stress. This could be either active (walking) or passive (sitting), but both active and passive behavior involved noticing natural surroundings and was to be performed for stress reduction. The instrument (with scoring) for this study was designed to assess intentional outdoor nature contact using the constructs of multi-theory model (MTM) of health behavior change. The instrument consisted of a total of 48 items and was validated by a panel of six experts in two rounds for face and content validity. There were 9 items capturing the participant demographics and 10 items based on the “Perceived Stress Scale (PSS-10)” [42], which was rated on a 5-point scale from never (0) to almost never (1), sometimes (2), fairly often (3), and very often (4). Four items were reverse coded. After they were scored, the scores of each item were summed and the possible minimum and maximum scores ranged from 0 to 40 units. 

Constitutively, the first construct in MTM is participatory dialogue, and this defined as convincing the participants about the possible advantages of behavior change exceeding the possible disadvantages [27,28]. Operationally, six items measured the “advantages of the participatory dialogue” construct (e.g., be healthier, be happier, not feel tired, etc.), and six items measured the “disadvantages of the participatory dialogue” construct (e.g., having less time for other things, finding it boring, finding it useless, etc.). The items were rated on a 5-point scale from never (0) to almost never (1), sometimes (2), fairly often (3), and very often (4). The score of participatory dialogue construct was obtained by subtracting the score of disadvantages from the advantages score (possible minimum and maximum scores ranged from −24 to +24 units). Constitutively, the second construct in MTM is behavioral confidence, which is defined as the futuristic confidence in an individual to make a behavior change that can have an internal or an external source [27,28]. Operationally, five items assessed the “behavioral confidence” construct (e.g., being able to start 20 min of intentional outdoor nature contact behavior daily, despite other duties, despite being busy, etc.) on a scale of not at all sure (0), slightly sure (1), moderately sure (2), very sure (3), completely sure (4), with possible minimum and maximum scores ranging from 0 to 20 units. Constitutively, the third construct in MTM is changes in the physical environment, which are defined as making modifications related to the availability and accessibility of resources that help in the initiation of the behavior change [27,28] Operationally, there were three items (finding a place, eliminating distractions, having necessary resources) on a scale of not at all sure (0), slightly sure (1), moderately sure (2), very sure (3), completely sure (4), with possible minimum and maximum scores ranging from 0 to 12 units that assessed the construct of “changes in the physical environment.” 

The fourth construct in MTM is the emotional transformation that entails converting emotions or feelings toward the goal of maintaining behavior change {27,28]. Operationally, three items (setting emotions to goals, self-motivation, and overcoming self-doubt) assessed the “emotional transformation” construct on a scale of not at all sure (0), slightly sure (1), moderately sure (2), very sure (3), completely sure (4), with possible minimum and maximum scores ranging from 0 to 12 units. The fifth construct in MTM is the practice for change, which entails constantly thinking about the behavior change and making mid-course rectifications to one’s strategy, overcoming barriers, and remaining focused on the behavior change to maintain behavior change [27,28]. Operationally, three items (keeping a self-diary/journal, overcoming barriers, and ability to change plans) measured the “practice for change” construct on a scale of not at all sure (0), slightly sure (1), moderately sure (2), very sure (3), completely sure (4), with possible minimum and maximum scores ranging from 0 to 12 units. The sixth construct in MTM is the change in the social environment, which is defined as developing social support from the environment to help with the maintenance of behavior change [26,27]. Operationally, three items (support from family, friends, and health professionals) assessed the construct of “changes in the social environment” on a scale of not at all sure (0), slightly sure (1), moderately sure (2), very sure (3), completely sure (4), with possible minimum and maximum scores ranging from 0 to 12 units. 

Finally, practicing intentional outdoor nature contact for 20 min daily in the upcoming week (initiation) and practicing intentional outdoor nature contact for 20 min daily for the next five years (sustenance) were measured on a scale of not at all likely (0), somewhat likely (1), moderately likely (2), very likely (3), and completely likely (4). For internal consistency, Cronbach’s alphas were calculated for all subscales and the entire scale (which was constituted of all subscales), and a confirmatory factor analysis was performed for construct validation.

### 2.4. Ethical Approval

The approval for this study was obtained from the Institutional Review Board (IRB) at the university. Data were collected only after IRB approval (approved by University of North Florida IRB under Protocol # 1218633-2). A statement in the online survey tool, directions indicating the nature of participation as voluntary, having no direct benefits, and the maintenance of confidentiality by researchers served as informed consent for completing the survey. 

### 2.5. Data Analyses

Data were analyzed for descriptive and inferential statistics using SPSS version 25. For descriptive statistics, frequencies and percentages were calculated for all the demographics and study variables that were categorical. Means and standard deviations were reported for those variables that were continuous. To establish the internal consistency of the scale, Cronbach’s alphas were calculated for each subscale and the entire scale. For construct validation of the scale, a confirmatory factor analysis using the maximum likelihood method was employed. Stepwise multiple regression modeling was performed for both the initiation model consisting of participatory dialogue, behavioral confidence, and changes in the physical environment as predictors; and for the sustenance model with emotional transformation, practice for change, and changes in the social environment as predictors. 

## 3. Results

A total of 401 students consented to participate in this study, of which 257 (64.1%) reported that during the past 24 h, they did not engage in intentional outdoor nature contact for stress reduction. Of 401 respondents, 281 (70.1%) reported that over the past 7 days, they practiced less than 20 min of intentional outdoor nature contact for stress reduction on average each day and were included as participants, which satisfied the sample size requirement for the current study.

The mean age of the study participants was 21.69 (±6.39) years. About 60% were female, 58% were White or Caucasian American, 6.8% had completed college or had a graduate degree, and 56.9% reported working for pay. Regarding the current financial situation, 50.2% indicated having enough money to pay their bills. Detailed results of the socio-demographic characteristics of the study participants are depicted in Table 1. 

The mean score for perceived stress was 21.60 (SD: ±7.08, possible minimum and maximum scores ranging from 0 to 40). Perceived stress was not statistically significantly correlated with the initiation and sustenance of practicing intentional outdoor nature contact. The mean for the initiation of practicing intentional outdoor nature contact for 20 min daily in the upcoming week was 1.07 (SD: ±1.19, with possible minimum and maximum scores ranging from 0 to 4). The mean for the sustenance of practicing intentional outdoor nature contact for daily for the next five years was 0.95 (SD: ±1.10, with possible minimum and maximum scores ranging from 0 to 4). Additional descriptive statistics of MTM constructs are depicted in Table 2. As can be seen from Table 2, all the scales and subscales had Cronbach’s alphas higher than 0.70, indicating they were acceptable [43]. The construct validation also yielded one-factor solutions for each subscale, with Eigenvalues over 1.0 and factor loadings over 0.32.

The results of the correlations and stepwise multiple regression for the initiation model are depicted in Table 3 and Table 4, respectively. The variance inflation factor (VIF) values were less than 10, indicating no multicollinearity in the regression analysis [44]. The model explained 57.5% of the variance in the initiation of intentional outdoor nature contact, *F* (2, 205) = 140.864, *p* < 0.001, adjusted *R*^2^ = 0.575. Behavioral confidence (standardized coefficient = 0.591, *p* < 0.001) and changes in the physical environment (standardized coefficient = 0.271, *p* < 0.001) statistically significantly predicted the initiation of intentional outdoor nature contact behavior. 

The dependent variable is the initiation of intentional outdoor nature contact; the independent variables are behavioral confidence and changes in the physical environment; *B* = unstandardized coefficient; *SE_B_* = standard error of the coefficient; *β* = standardized coefficient; *p* = level of significance; *CI* = confidence interval; participatory dialogue (advantages/disadvantages) was excluded from the model (probability of F to remove ≥0.1).

The results of the correlations and stepwise multiple regression for the sustenance model are depicted in Table 5 and Table 6, respectively. The VIF values were less than 10, indicating no multicollinearity in the regression analysis [44]. The model explained 31.0% of the variance in the sustenance of intentional outdoor nature contact, *F* (3, 214) = 33.427, *p* < 0.001, adjusted *R*^2^ = 0.310. Emotional transformation (standardized coefficient = 0.173, *p* = 0.021), practice for change (standardized coefficient = 0.317, *p* < 0.001), and changes in the social environment (standardized coefficient = 0.204, *p* = 0.002) statistically significantly predicted the sustenance of intentional outdoor nature contact behavior.

The dependent variable is the sustenance of intentional outdoor nature contact; the independent variables are emotional transformation, practice for change, and changes in the social environment; *B* = unstandardized coefficient; *SE_B_* = standard error of the coefficient; *β* = standardized coefficient; *p* = level of significance; *CI* = confidence interval.

## 4. Discussion

The purpose of this study was to identify the determinants of intentional outdoor nature contact behavior utilizing the tenets of the multi-theory model (MTM) of health behavior change. Based on the study, it was found that behavioral confidence (*p* < 0.001) and changes in the physical environment (*p* < 0.001) were statistically significant explanatory constructs for the initiation of intentional outdoor nature contact behavior among college students and together accounted for a substantial proportion of variance (57.5%). In behavioral and social sciences, such magnitude is of practical significance. Behavioral confidence [30,31,32,33,34,35,36,37] and changes in the physical environment [31,35,36,37] have been found to be significant and substantial predictors of other health behaviors in several studies based on MTM with US college students. Participatory dialogue was not found to contribute to initiating intentional outdoor nature contact behavior. One possible explanation of this finding could be because the mean score for participatory dialogue was 3.92 (8.67) units, with possible minimum and maximum scores ranging from −24 to +24 units. While it was positive, it was still very low and, in the absence of an intervention, it probably did not reach the threshold to have any influence on intentional outdoor nature contact behavior. It could also be that college students were not convinced that this is a beneficial behavior and thus this construct did not account for a significant contribution to the dependent variable.

In this study, for maintaining intentional outdoor nature contact behavior, all the three constructs of MTM—namely, emotional transformation (*p* = 0.021), practice for change (*p* < 0.001), and changes in the social environment (*p* = 0.002)—were statistically significant and contributed substantively toward the variance (31.0%). Emotional transformation [30,31,32,33,34,35,37], practice for change [30,31,35,36,37], and changes in the social environment [30,31,34,37] have been found to be significant and substantial predictors of other health behaviors in several studies based on MTM with US college students.

As part of its secondary purpose, this study found that a large proportion of college students (64.1%) did not engage in intentional outdoor nature contact for stress reduction in the past 24 h. Further, 70.1% reported that over the past 7 days they had practiced less than 20 min of intentional outdoor nature contact for stress reduction on average each day. These findings are especially relevant, since the mean score for perceived stress was 21.60 (7.08) units on a scale of 0–40 units, or slightly above the mid-point. The data were collected in the month of October, and thus the stress due to examinations would perhaps not have played an important role and could be indicative of the general levels of stress experienced by college students. College students experience stress due to relationships, studies, daily hassles, boredom (or nonevents), work, and other such stressors [45]. When stress levels are high, the role of stress management programs becomes particularly valuable. Experimentation with theory-based health promotion interventions that promote intentional outdoor nature contact behavior among college students must be carried out. MTM provides a practical and useful framework for designing such interventions.

The findings of this study have important implications for designing MTM-based health promotion interventions that promote intentional outdoor nature contact behavior among college students. The advantage of using MTM is that it can deliver brief and precise interventions and can be easily linked to technological advancements, a trend that is emerging in the field of health promotion. The term precise intervention refers to the focused delivery of messages based on the significant and proven constructs of MTM which emphasize salient aspects of behavior change. In order to build behavioral confidence, the program can utilize mobile phone apps that provide cues for the initiation of intentional outdoor nature contact behavior. The program can provide small steps for indulging in this behavior that can lead to mastery of the skills pertaining to the appreciation of nature while walking (active) or sitting (passive). This can help reduce the stress associated with daily stressors. For building the construct of changes in the physical environment, the health promotion strategies of cultivating land for nature appreciation, developing healing gardens, providing green space (parks, gardens, etc.) in communities, and advocating for the preservation of wilderness are some strategies that can go a long way [4]. For example, Cornell University has developed a “Nature Prescription Program”, whereby the university community works to enhance the natural beauty of their campus so that it positively impacts every member [46].

For developing the construct of emotional transformation, the participants in the health promotion program must be taught techniques to convert their feelings into goals. For example, students when feeling frustrated or angry instead of indulging in that feeling they can make a goal to take a stroll in the park and admire nature. Not only will that help them get diverted from negative feelings, but it will enable them to relax and manage stress effectively. For influencing the construct of practice for change one must constantly be thinking and reminding oneself of the goal to engage in the behavior of intentional outdoor nature contact. Once again, technology can help this process. The use of text messaging reminders or mobile phone apps can be helpful. Finally, for modifying the construct of changes in the social environment, the program personnel can mobilize either natural (such as through friends, peers, and family) or artificial (such as through health professionals or researchers) social support.

### Limitations of the Study

The study had some limitations. First, the study used a cross-sectional design, which is a snapshot in time. The weakness of this design is the lack of ability to establish a temporal relationship, which, though vital for causal inference [47], has been recently debated [48]. We chose this study design for practical reasons and future studies can use more robust longitudinal designs. Second, the study relied on self-reports which can lead to measurement error in the form of dishonest responses, bias, exaggeration, etc. While attitudinal variables can only be measured through self-report, the actual performance of the behavior can be objectively observed but is usually not feasible as was the case in this study. Future studies must attempt to measure actual behavior objectively. Third, the study used intentional outdoor nature contact behavior for both initiation and sustenance models which is only a proxy of the actual behavior. Finally, the study did not establish the test-retest (stability) reliability of the instrument used. This was due to lack of adequate resources. Future studies must attempt to do that.

## 5. Conclusions

Intentional outdoor nature contact behavior is an untapped stress alleviator that can be leveraged in stress management programs for college students. The contemporary, fourth-generation multi-theory model (MTM) of health behavior change provides a useful and pragmatic framework for designing such interventions. The findings of this study lend credence to the hypothesis that the constructs of this model are significant and contribute to a substantial proportion in explaining the variance in the intentional outdoor nature contact behavior. Future research must operationalize MTM in designing and evaluating health promotion interventions that promote intentional outdoor nature contact behavior.

## Figures and Tables

**Table 1:** Socio-demographic characteristics of the participants (*n* = 281).

Characteristic	Mean (SD)	*n* (%)
**Age (years)**	21.69 (±6.39)	
**Gender**		
Male		82 (29.2%)
Female		167 (59.4%)
Other		6 (2.1%)
**Race/Ethnicity**		
White or Caucasian American		163 (58.0%)
Black or African American		32 (11.4%)
Asian American		18 (6.4%)
American Indian		2 (0.7%)
Hispanic American		24 (8.5%)
Other		15 (5.3%)
**Education**		
Some schooling but not completed high school		1 (0.4%)
Completed high school or GED		27 (9.6%)
Some college		206 (73.3%)
Completed college/graduate degree		19 (6.8%)
Postgraduate degree		2 (0.7%)
**Work Status**		
Yes		160 (56.9%)
No		94 (33.5%)
**Current Financial Situation**		
I do not have enough money to pay my bills each month		44 (15.7%)
I have enough money to pay my bills		141 (50.2%)
I have enough money to pay my bills and some left over		64 (22.8%)
**Current situation (If you were to receive an unexpected $500 medical bill, would you…)**		
Be able to pay the bill with no problem		73 (26.0%)
Be able to pay the bill on a monthly basis		114 (40.6%)
Not be able to pay the bill		65 (23.1%)

Note: The characteristic categories are bolded for clarity. Due to missing data, the percentage of participants in each category of sociodemographic characteristics do not sum to 100%.

**Table 2:** Descriptive statistics of perceived stress and constructs of multi-theory model (MTM) of health behavior change (*n* = 281).

Constructs	Possible Min. & Max.	Observed Min. & Max.	Mean (SD)	Cronbach’s Alpha
Perceived Stress	0–40	4–38	21.60 (±7.08)	0.88
Initiation	0–4	0–4	1.07 (±1.19)	---
Participatory dialogue: advantages	0–24	0–24	15.07 (±4.84)	0.89
Participatory dialogue: disadvantages	0–24	0–24	11.20 (±4.90)	0.84
Participatory dialogue: advantages/disadvantages score	−24–+24	−19–+24	3.92 (±8.67)	---
Behavioral confidence	0–20	0–20	3.80 (±4.53)	0.92
Changes in the physical environment	0–12	0–12	5.23 (±3.64)	0.87
Entire initiation scale (advantages, disadvantages, behavioral confidence, and changes in the physical environment)	---	---	---	0.68
Sustenance	0–4	0–4	0.95 (±1.10)	---
Emotional transformation	0–12	0–12	4.17 (±3.23)	0.90
Practice for change	0–12	0–12	2.63 (±2.85)	0.81
Changes in the social environment	0–12	0–12	3.99 (±3.29)	0.76
Entire sustenance scale (emotional transformation, practice for change, changes in the social environment)	---	---	---	0.87
Entire scale	---	---	---	0.86

**Table 3:** Correlation matrix of the initiation model constructs.

Construct	1	2	3	4
1. Initiation	-	0.470 **	0.695 **	0.570 **
2. Participatory dialogue: advantages/disadvantages score		-	0.473 **	0.414 **
3. Behavioral confidence			-	0.488 **
4. Changes in the physical environment				-

** Correlation is significant at the 0.01 level.

**Table 4:** Stepwise multiple regression predicting initiation for intentional outdoor nature contact (*n* = 281).

Variables	*B*	*SE_B_*	*β*	*p*-Value	95% *CI*
Behavioral confidence	0.156	0.014	0.591	<0.001	0.129, 0.182
Changes in the physical environment	0.089	0.017	0.271	<0.001	0.056, 0.123

F (2, 205) = 140.864, *p* < 0.001, R^2^ = 0.579, adjusted R^2^ = 0.575.

**Table 5:** Correlation matrix of the sustenance model constructs.

Construct	1	2	3	4
1. Sustenance	-	0.450 **	0.480 **	0.396 **
2. Emotional Transformation		-	0.603 **	0.451 **
3. Practice for Change			-	0.376 **
4. Changes in the Social Environment				-

** Correlation is significant at the 0.01 level.

**Table 6:** Stepwise multiple regression predicting the sustenance of intentional outdoor nature contact (*n* = 281).

Variables	*B*	*SE_B_*	*β*	*p*-Value	95% *CI*
Emotional Transformation	0.059	0.025	0.173	0.021	0.009, 0.108
Practice for Change	0.124	0.028	0.317	<0.001	0.069, 0.179
Changes in the Social Environment	0.068	0.021	0.204	0.002	0.026, 0.110

F (3, 214) = 33.427, *p* < 0.001, R^2^ = 0.319, adjusted R^2^ = 0.310.

## Data Availability

The datasets used and/or analyzed during the current study are available from the corresponding author on reasonable request.
